# Progressive pulmonary fibrosis: current perspectives in diagnostic imaging

**DOI:** 10.1093/bjro/tzaf018

**Published:** 2025-07-24

**Authors:** Prerana Agarwal, Christopher L Schlett, Fabian Bamberg, Björn C Frye

**Affiliations:** Department of Diagnostic and Interventional Radiology, Medical Center—University of Freiburg, Faculty of Medicine, University of Freiburg, 79106 Freiburg im Breisgau, Germany; Department of Diagnostic and Interventional Radiology, Medical Center—University of Freiburg, Faculty of Medicine, University of Freiburg, 79106 Freiburg im Breisgau, Germany; Department of Diagnostic and Interventional Radiology, Medical Center—University of Freiburg, Faculty of Medicine, University of Freiburg, 79106 Freiburg im Breisgau, Germany; Department for Pneumology, Medical Center—University of Freiburg, Faculty of Medicine, University of Freiburg, 79106 Freiburg im Breisgau, Germany

**Keywords:** pulmonary fibrosis, interstitial lung diseases, multidetector computed tomography, hypersensitivity pneumonitis, sarcoidosis

## Abstract

A subset of patients with interstitial lung diseases (ILDs) experiences disease progression despite standard treatment protocols. Similar to idiopathic pulmonary fibrosis, the archetype of progressive fibrotic ILDs, these patients exhibit worsening clinical symptoms, declining lung function, and progressive radiological changes, often resulting in shortened survival. This progressive disease pattern is classified under the term progressive pulmonary fibrosis or progressive fibrosing ILD. Radiological imaging, particularly high-resolution computed tomography (HRCT), is integral to diagnosing ILDs and plays a critical role within multidisciplinary ILD boards. HRCT is instrumental in identifying patients at a higher risk for disease progression and may provide valuable prognostic insights. Additionally, serial imaging is essential for detecting progression over time. While visual assessment remains the primary method for evaluating disease advancement, emerging quantitative techniques, including those utilizing machine learning, are currently undergoing validation.

## Introduction

Interstitial lung diseases (ILDs) are a group of diffuse parenchymal lung diseases characterized by various degrees of inflammation and fibrosis in the distal bronchoalveolar space.^1^ Among them, fibrotic ILDs account for a significant burden of mortality and morbidity. Idiopathic pulmonary fibrosis (IPF) is the archetype of chronic fibrosing ILDs, which typically shows a progressive phenotype characterized by worsening of symptoms, deteriorating lung function, and early death. Similar to IPF, some other fibrotic ILDs show a tendency to progress despite standard treatment protocols. Consequently, ILDs exhibiting this progressive phenotype are grouped under the term progressive pulmonary fibrosis (PPF).^2^

The aim of this narrative review is to provide an overview of the current definition of PPF and highlight common risk factors associated with disease progression. The primary focus is on the radiological assessment of PPF in routine clinical practice, with practical guidance, tips, and illustrative examples to support diagnostic accuracy. In addition to a qualitative visual evaluation, the review includes a concise summary of emerging quantitative imaging approaches relevant to the evaluation of disease progression.

## Progressive pulmonary fibrosis: definition

Many patients with non-IPF fibrotic lung diseases show disease stabilization or improvement with treatment. For example, those with connective tissue disease-related ILD (CTD-ILD) and idiopathic nonspecific interstitial pneumonia (iNSIP) often improve with immunosuppressive medications. Similarly, patients with fibrotic hypersensitivity pneumonitis (f-HP) potentially benefit from eliminating exposure to triggering antigens. However, some patients continue to experience worsening disease despite receiving appropriate treatment. This pattern of ongoing deterioration is known as PPF or progressive fibrosing ILD (PF-ILD). It includes various types of fibrotic ILDs (F-ILDs) that progress similarly to IPF.

The current consensus-based definition of PPF was first published in the 2022 update of the ATS/ERS/JRS/ALAT guidelines. The criteria are derived from various clinical trials and are summarized in Table 1.^2^ In simplified terms, PPF refers to a non-IPF ILD with radiological signs of fibrosis on high-resolution computed tomography (HRCT) (honeycombing and traction bronchiectasis) and evidence of progression over time. Progression is defined as the presence of at least 2 of the following 3 criteria within the previous year: worsening respiratory symptoms, lung functional decline, and radiological progression. It is important to exclude alternative explanations for worsening features in patients with suspected progression. Although the international guideline criteria for PPF do not require patients to demonstrate progression despite treatment, various consensus statements recommend incorporating the phrase “despite adequate or usual management” into the definition of PPF.^3–5^ Moreover, while the guideline defines a 12-month period for a PPF diagnosis, meaningful progression may occur over shorter or longer durations, and it has been argued that progression should be recognized as such, regardless of timing.^6^

**Table 1.** Definition of progressive pulmonary fibrosis (PPF) as defined by the international guideline idiopathic pulmonary fibrosis (an update) and progressive pulmonary fibrosis in adults by ATS/ERS/JRS/ALAT (adapted from ref.^2^).

In a patient with ILD other than IPF with radiological evidence of pulmonary fibrosis: At least 2 of the following 3 criteria occurring within the past year with no alternative explanation	
1.	Worsening respiratory symptoms
2.	Physiological evidence of disease progression (either of the following):
	Absolute decline in FVC > 5% predicted within 1 year of follow-up
	Absolute decline in DLCO(corrected for Hb)>10% predicted within 1 yr of follow-up
3.	Radiological evidence of disease progression (at least one or more of the following):
	Increased extent or severity of traction bronchiectasis and bronchiolectasis
	New ground-glass opacity with traction bronchiectasis
	New fine reticulation
	Increased extent or increased coarseness of reticulations
	New or increased honeycombing
	Increased lobar volume loss

It is important to understand that PPF is not a separate diagnosis on its own; instead, it describes a pattern of disease behaviour that can occur in patients with F-ILDs. Even though PPF is a clinical phenotype, every effort should still be made in providing a disease-specific multidisciplinary diagnosis to enable specific measures, such as investigating a possible provoking agent in the case of HP or immunomodulatory therapies in the context of CTD-ILDs. An accurate initial diagnosis is also essential for proper risk stratification and understanding the likelihood of disease progression, as different fibrotic ILDs carry varying risks of progression. In this context, a recent study including patients with etiologically different NSIP (CTD-ILD and idiopathic) demonstrated that intensifying immunosuppression resulted in disease stabilization.^7^

It is important not to confuse PPF with a distinct entity known as progressive massive fibrosis (PMF). PMF is characterized by large, mass-like opacities, typically located in the upper lobes, and is seen in patients with pneumoconiosis, such as coal workers’ pneumoconiosis or silicosis.^8^

## Prevalence and risk factors

**Figure 1.**
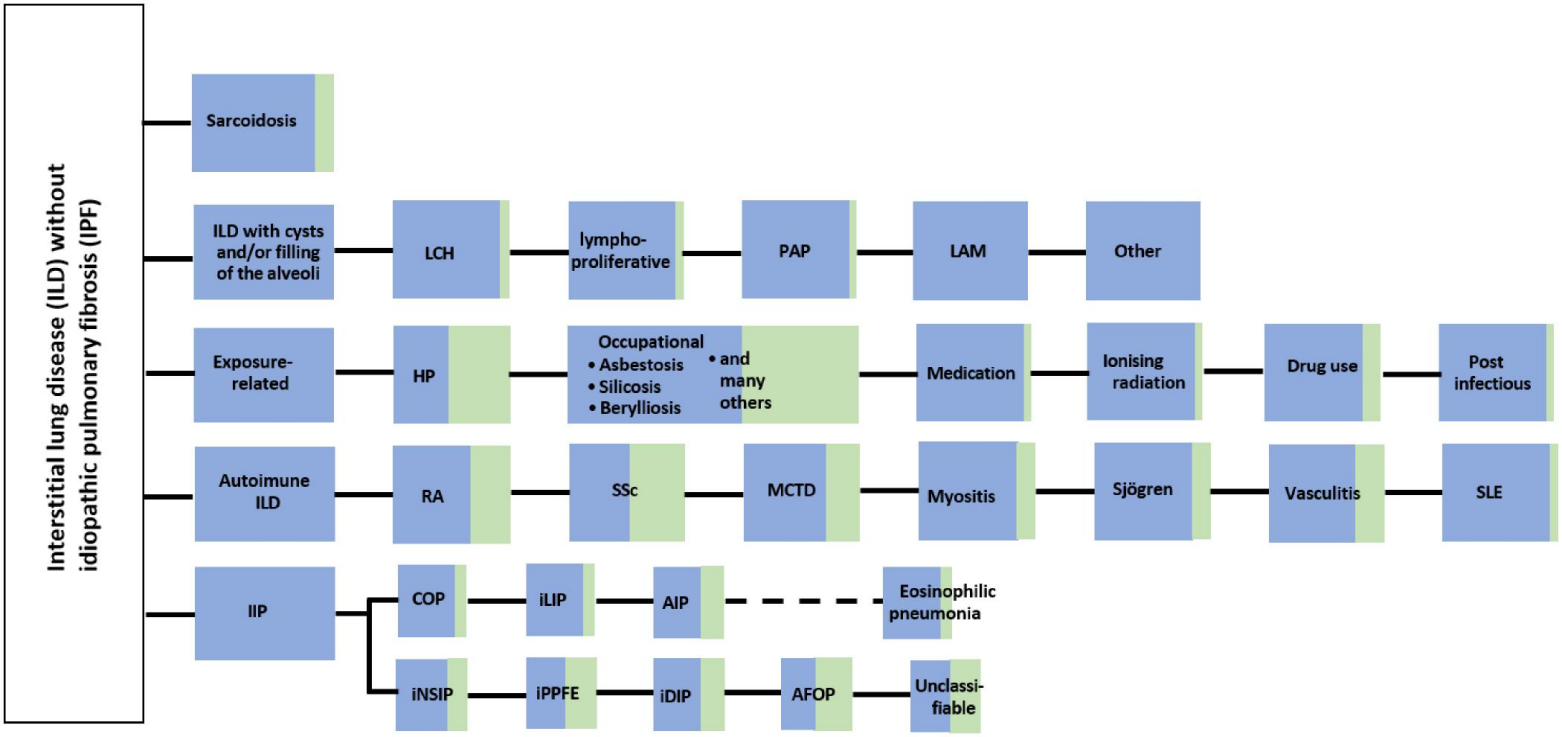
Interstitial lung diseases (ILDs) without idiopathic pulmonary fibrosis (IPF) that can show progressive pulmonary fibrosis (PPF) (adapted from refs^2^^,^^5^). The green area depicts an estimated proportion of patients with various types of ILD who can show PPF. Abbreviations: AFOP = acute fibrinous and organizing pneumonia; AIP = acute interstitial pneumonia; COP = cryptogenic organizing pneumonia; DM = dermatomyositis; HP = hypersensitivity pneumonitis; iDIP = idiopathic DIP; IIP = idiopathic interstitial pneumonia; iLIP = idiopathic lymphoid interstitial pneumonia; iNSIP = idiopathic nonspecific interstitial pneumonia; iPPFE = idiopathic pleuroparenchymal fibroelastosis; LAM = lymphangioleiomyomatosis; LCH = Langerhans cell histiocytosis; MCTD = mixed connective tissue disease; PAP = pulmonary alveolar proteinosis; PM = polymyositis; RA = rheumatoid arthritis; SLE = systemic lupus erythematosus; SSc = systemic sclerosis.

It is estimated that 18%-32% of non-IPF ILDs progress despite initial treatment.^4^^,^^9^^,^^10^ There is a wide variation in the estimated incidence, mainly resulting from variation in study design, geographical differences, and different definitions of progression used. Common underlying ILD diagnoses with a progressive fibrosing phenotype include unclassified ILD (17.2%-71.4%), autoimmune ILDs (16.6%-46.7%), f-HP (1.6%-40.0%), other F-ILDs (exposure-related ILD, sarcoidosis, and other fibrosing ILD) (6.1%-36.4%) and iNSIP (0.7%-32.3%)^11^ (Figure 1). Other conditions with an increased risk for PPF include pleuroparenchymal fibroelastosis, fibrosing organizing pneumonia, desquamative interstitial pneumonia, fibrotic occupational ILD and fibrotic Langerhans cell histiocytosis.^12^ Each ILD shows varying risk of progression. For example, risk of progression is higher in patients with unclassified ILD.^4^^,^^13^ Furthermore, the risk of progression is higher in f-HP when the inciting antigen cannot be identified.^14^ Thus, an accurate initial diagnosis is pivotal to predict the risk of progression despite management.

Generalizable risk factors for disease progression include advanced age, genetic risk factors such as short telomere syndrome and *MUC5B* promoter polymorphism, and absence of response to treatment.^15^ Lower baseline lung function in certain ILDs like f-HP and systemic sclerosis-associated ILD and smoking history in RA-ILD have been shown to be associated with a higher risk of progression.^4^ In patients with systemic sclerosis-associated ILD, gastro-oesophageal reflux disease is associated with progressive fibrosis because of repeated microaspirations.^16^ A coexisting pleuroparenchymal fibroelastosis in patients with other ILDs is associated with progressive decline in lung function and poor prognosis.^4^^,^^17^ On the contrary, it has been shown that progression can be slower in certain CTD-ILDs, like the idiopathic inflammatory myositis-ILD subtype.^4^^,^^18^

### Radiological risk factors for progression

It is difficult to predict the proportion of patients with non-IPF ILDs who will develop a progressive fibrotic pattern; however, some HRCT findings are considered predictive of disease progression. A major radiological risk factor for PPF is radiological usual interstitial pneumonia (UIP) pattern, regardless of a specific ILD diagnosis. For example, in patients with rheumatoid arthritis-related ILD (RA-ILD) and f-HP, the UIP pattern is known to be associated with poor prognosis.^19^^,^^20^ This may be related to shared genetic risk factors such as *MUC5B* promoter polymorphism.^21^ Presence of honeycombing and traction bronchiectasis are known to be associated with worse prognosis.^22^^,^^23^ A greater extent of fibrotic changes is known to be predictive of mortality in IPF, RA-ILD, systemic sclerosis-related ILD, f-HP, pulmonary sarcoidosis, and unclassified ILDs.^24^

## Diagnostic approach

Early diagnosis of PPF is essential to facilitate timely and appropriate treatment. Monitoring disease progression in F-ILD involves a comprehensive approach that combines clinical symptom assessment, pulmonary function tests (PFTs), and HRCT imaging. Multidisciplinary team (MDT) discussions are now essential not only for the initial diagnosis of ILDs but also for follow-up, enabling evaluation of disease trajectory and early identification of patients with PPF.^25^

Although there are no universally accepted guidelines for the frequency of pulmonary function tests (PFTs) due to the variable disease course among patients, expert consensus recommends conducting PFTs every 3-4 months during the first year following an F-ILD diagnosis.^3^^,^^4^ There is no recommendation to suggest routine follow-up HRCT to evaluate for signs of progression, and imaging is indicated when there is clinical suspicion of worsening of fibrosis or when serial pulmonary function data are inconclusive. Some studies have shown that in patients with systemic sclerosis and stable pulmonary function, repeated chest HRCT within 12-24 months from baseline could be useful to promptly detect radiological progression.^26^

From a radiological standpoint, while it is important to describe radiological signs of progression in our reports, the diagnosis of PPF is best made in the context of an MDT discussion. This distinction is crucial in clinical practice.

## Radiological assessment of PPF

### Visual assessment

Progression of fibrosis is typically assessed visually by comparing similar HRCT slices of the initial and follow-up CT examinations side by side. Progress can be seen as a quantitative change in follow up CTs and/or as a qualitative change. A quantitative progression in this context signifies an increased extent of pre-existing fibrosis, for example, extent and number of honeycombing cysts or extent of reticulation in a lobe. In contrast, qualitative progression means a change in the type of interstitial alterations, for example, ground-glass opacities that progress to reticulations or increased coarseness of reticulation. New development of honeycombing reflecting a change from a probable UIP pattern to a definite UIP pattern also signifies progress.

**Figure 2.**
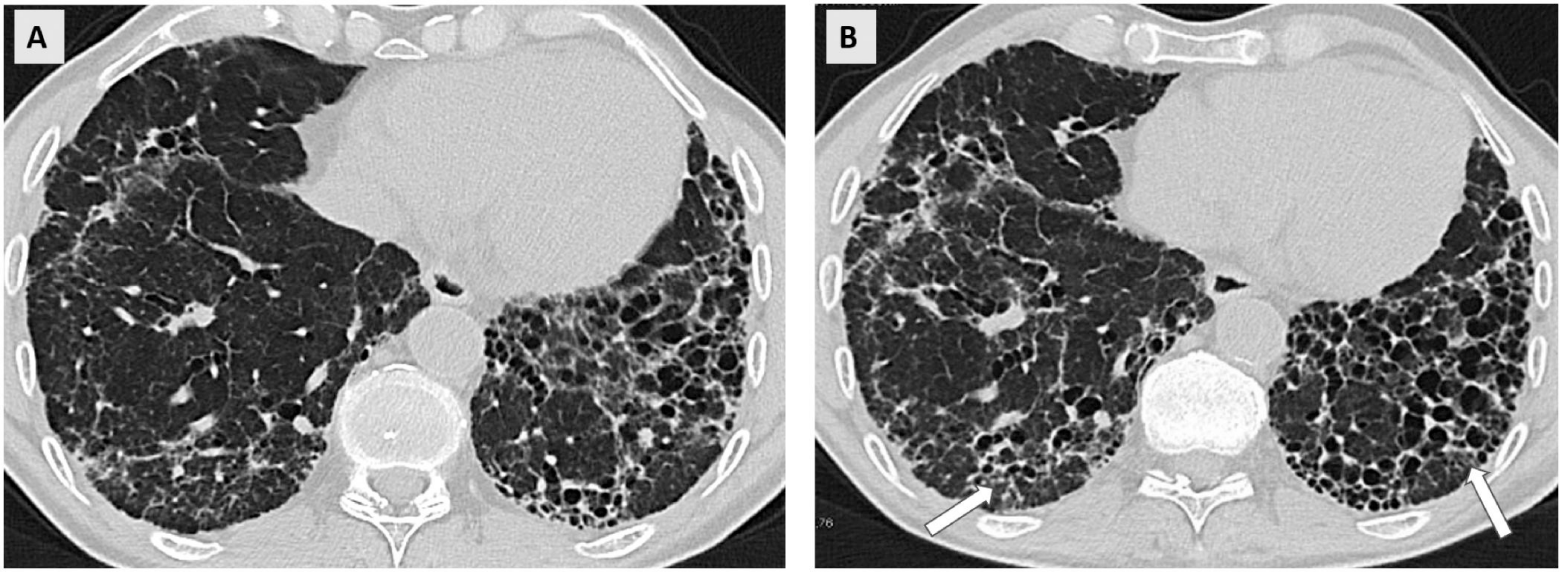
Progressive Pulmonary Fibrosis in a patient with fibrotic hypersensitivity pneumonitis. (A) Axial CT scan of the lower lobes from December 2023. (B) Comparable axial section from a follow-up CT scan obtained in November 2024 due to worsening symptoms showing signs of progressive fibrosis with progressive traction bronchiectasis (arrows) and reticulations.

Some patients with acute non-fibrotic HP can develop f-HP, and some of these patients develop a progressive fibrosing phenotype which is associated with a worse outcome. The radiological diagnosis of non-fibrotic HP requires at least one HRCT abnormality indicative of parenchymal infiltration (ground-glass opacities or mosaic attenuation), and at least one HRCT abnormality indicative of small airways disease (air trapping or centrilobular ground glass nodules), both in a diffuse distribution.^27^ In patients with f-HP, additional signs of fibrosis are evident, which may cause the 3-density pattern. Increase in the extent of findings on CT signifies a radiological progression (Figure 2). Additionally, a change of disease stage from non-fibrotic to f-HP qualifies as progress, with progressive signs of fibrosis such as traction bronchiectasis and the development of honeycombing.

**Figure 3.**
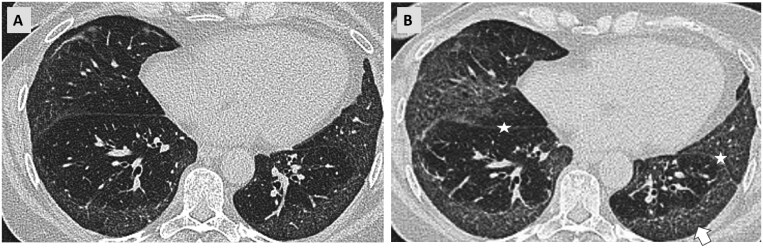
Progressive signs of fibrosis in a patient with Sjögren’s syndrome and pulmonary involvement showing an NSIP pattern. (A) Axial CT scan of the lower lobes from 2020 showing fine reticulations and ground-glass opacities with subpleural sparing. (B) Comparable axial section from a follow- up CT scan obtained in 2023 shows progressive fibrosis with progressive reticulations, emerging traction bronchiectasis (arrow) and progressive volume loss of the lower lobes as observed by the dorsal displacement of the fissures (stars).

NSIP is a pattern of lung fibrosis that is typically seen in younger female patients and is more often associated with connective tissue diseases. Progression in NSIP can manifest as an increase in the extent of lung parenchymal involvement, which can be assessed visually (Figure 3). Additionally, a change from cellular NSIP (predominantly subpleural ground-glass opacities) towards a mixed cellular-fibrotic or a predominantly fibrotic NSIP (basal and bronchocentric traction bronchiectasis and reticulations) or the development of a UIP pattern also signifies progression.^28^

**Figure 4.**
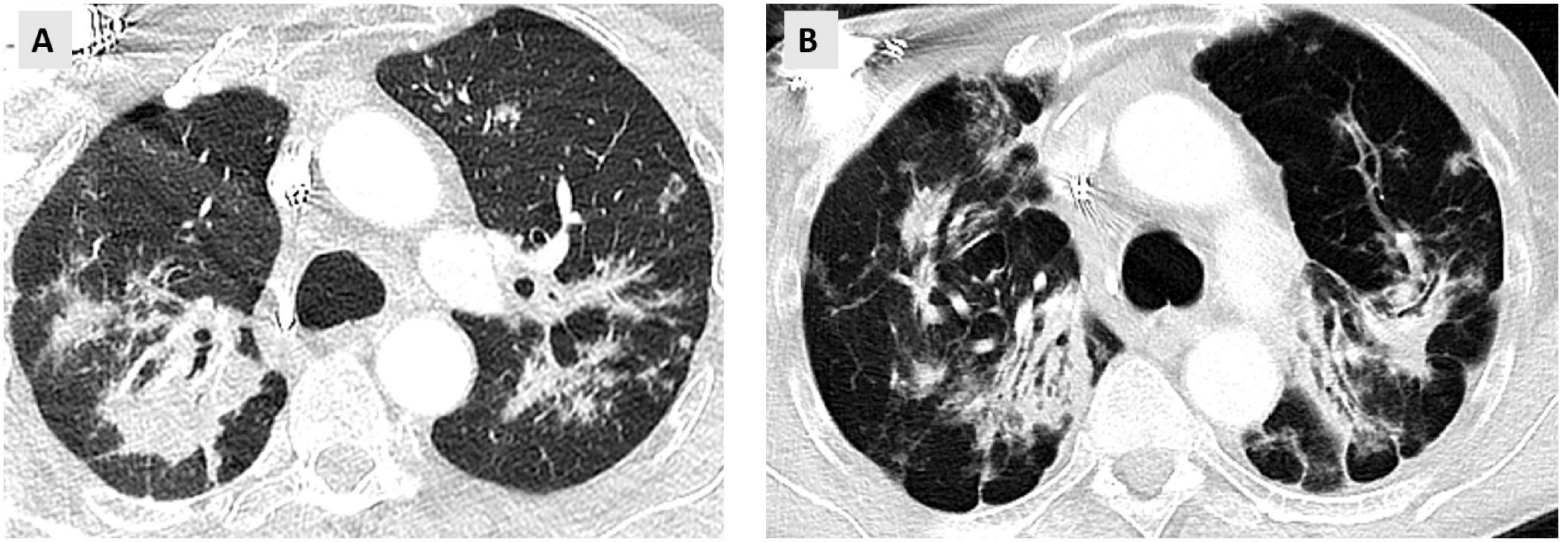
Axial CT scan sections through the upper lobes of a patient with pulmonary sarcoidosis. (A) CT image from 2016 showing a typical pattern with perilymphatic nodules in the left upper lobe and a predominant bronchocentric consolidation in the right upper lobe. (B) Follow-up CT a few years later shows signs of progressive fibrosis with progressive volume loss and posterior distortion of the upper lobe bronchi.

Patients with sarcoidosis can evolve from non-fibrotic sarcoidosis (predominantly nodular pattern) to a fibrotic phenotype. Typically, fibrotic sarcoidosis shows involvement of posterior upper lobes with progressive volume loss, distortion of the upper lobe bronchus more posteriorly, and tenting of the diaphragm (Figure 4).

It needs to be emphasized that the radiological criteria for progress have not been validated in larger studies. Visual assessment of HRCT scans leaves room for intra- and interreader variability.^29^

## Tips and tricks

Patients who develop PPF can show various degrees of deterioration, with some patients developing rapidly progressive changes, both clinically and radiologically. Other patients might show a less dramatic progression clinically, with the only subtle signs of progression being notable radiologically. Some tips and tricks that could be helpful in the clinical routine for identifying radiological PPF are as follows:

*Image interpretation based on clinical scenario:* It is of utmost importance to evaluate the follow-up scans in light of the clinical symptoms. In patients suspected of having an acute exacerbation due to infection or left heart failure, any new or progressive consolidations, ground-glass opacities, and reticulations should be judged with caution with regard to progression. CT pulmonary angiography should be considered when considering pulmonary embolism in the differential diagnosis. In case of equivocal findings, it may be worthwhile to perform a repeat CT scan after the acute episode to confirm or refute the suspicion of progression.

*Comparison of scans with different scan parameters:* Care should be taken while comparing follow-up HRCT scans performed on different scanners with different parameters such as reconstruction thickness. Thinner slices on follow-up can mimic progressive reticulations due to a better resolution of the opacities. Scans performed on the newer photon-counting scanners have shown enhanced image sharpness and improved visibility of ILD changes, which may seem like progress when compared to prior scans on conventional CT scanners and must be viewed with caution.^30^

*Attention to the acquisition method:* Volume acquisition of CT is now the recommended standard for the evaluation of ILDs. These datasets provide an added advantage over the spaced axial technique as they enable evaluation of the entire lung parenchyma and eliminate the risk of missing out on progressive changes in the interslice gaps. Another added benefit of these volume acquisitions is the possibility of multiplanar reconstructions. Comparing the lung parenchyma in follow-up scans, especially the fissures in coronal and sagittal reconstructions, can draw one’s attention to progressive traction bronchiectasis and progressive volume loss. These findings can sometimes be missed on axial scans.

**Figure 5.**
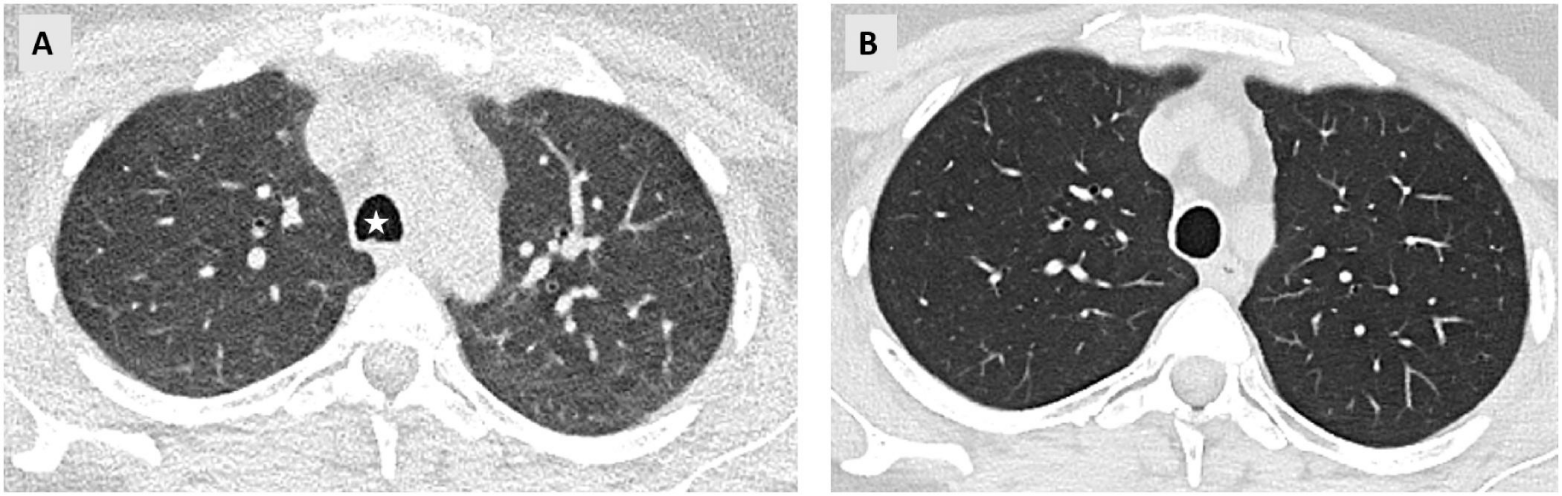
(A) Axial CT scan through the upper lobes showing diffuse ground-glass opacities. The posterior wall of the trachea shows a concave configuration (star) due to an expiratory scan. (B) Inspiratory CT scan with a rounded tracheal configuration showing well aerated lung parenchyma with no ground-glass opacities.

*Attention to patient factors:* Another important factor to consider while interpreting suspicious ground-glass opacities on follow-up scans is the patient′s compliance with breathing commands. Expiratory scans are associated with heterogeneous ground-glass opacities which are physiological to some extent. These changes can be falsely attributed to progressive fibrotic changes by inexperienced readers. A quick look at the tracheal configuration can resolve this dilemma in most cases, as the posterior tracheal wall shows a concave configuration in expiratory scans (Figure 5).

### Quantitative assessment: QCT

The traditional visual analysis of HRCT scans faces significant challenges: low sensitivity for changes over time and a high inter-observer variability.^31^ As treatment options for ILDs have expanded, research is increasingly focused on developing imaging biomarkers that could help predict disease progression and patient outcomes. Quantitative and computer-based analyses show promise in 2 key areas. First, they could enable more precise tracking of disease progression through identification of CT-based markers in scan comparisons over time.^32^^,^^33^ Second, and perhaps more importantly, these tools could identify patients at risk of progression based on their baseline scans alone. This predictive capability is especially valuable since current guidelines only allow anti-fibrotic treatment after progression has been documented—by which time the disease may be less responsive to intervention. Early identification of high-risk patients could enable more timely treatment when the disease course might be more effectively altered.^34^

In the last years, QCT has evolved from simple histogram-based measurements to more refined methods such as using machine learning and radiomics. In a whole lung histogram based approach, kurtosis has been shown to be predictive of short term mortality in patients with IPF.^35^

One study using an automated texture-based lung parenchymal characterization tool called CALIPER (Computer-Aided Lung Informatics for Pathology Evaluation and Rating) demonstrated that quantitative short-term changes in disease extent in serial CTs were predictive of survival.^36^ Subsequent studies using CALIPER showed that computer-derived fibrosis extent and other related features can play a prognostic role in various ILDs. For example, a CALIPER-derived feature “pulmonary vascular volume (PVV)” or “pulmonary vascular-related structures” was an important predictor of mortality in IPF, CTD-ILD and HP.^37–39^ Furthermore, an increase in PVV in serial imaging was shown to be the strongest CT determinant of lung function decline in IPF patients.^40^

One study involving 193 IPF patients demonstrated that a texture-based automated quantitative CT analysis could accurately predict stable lung function over 1 year when the extent of reticular opacities was less than 22.05%. Such tools have the potential to identify patients at a low risk of progression and tailor the follow-up strategy.^41^ Another machine-learning algorithm which quantifies the extent of reticulation and architectural distortion using a support vector machine classifier, known as QLF (quantitative lung fibrosis) was associated with baseline disease extent and a sensitive measure of lung function change over time.^42^ In systemic sclerosis-ILD, QLF scores correlate with changes in lung function and help quantify treatment response.^43^

**Figure 6.**
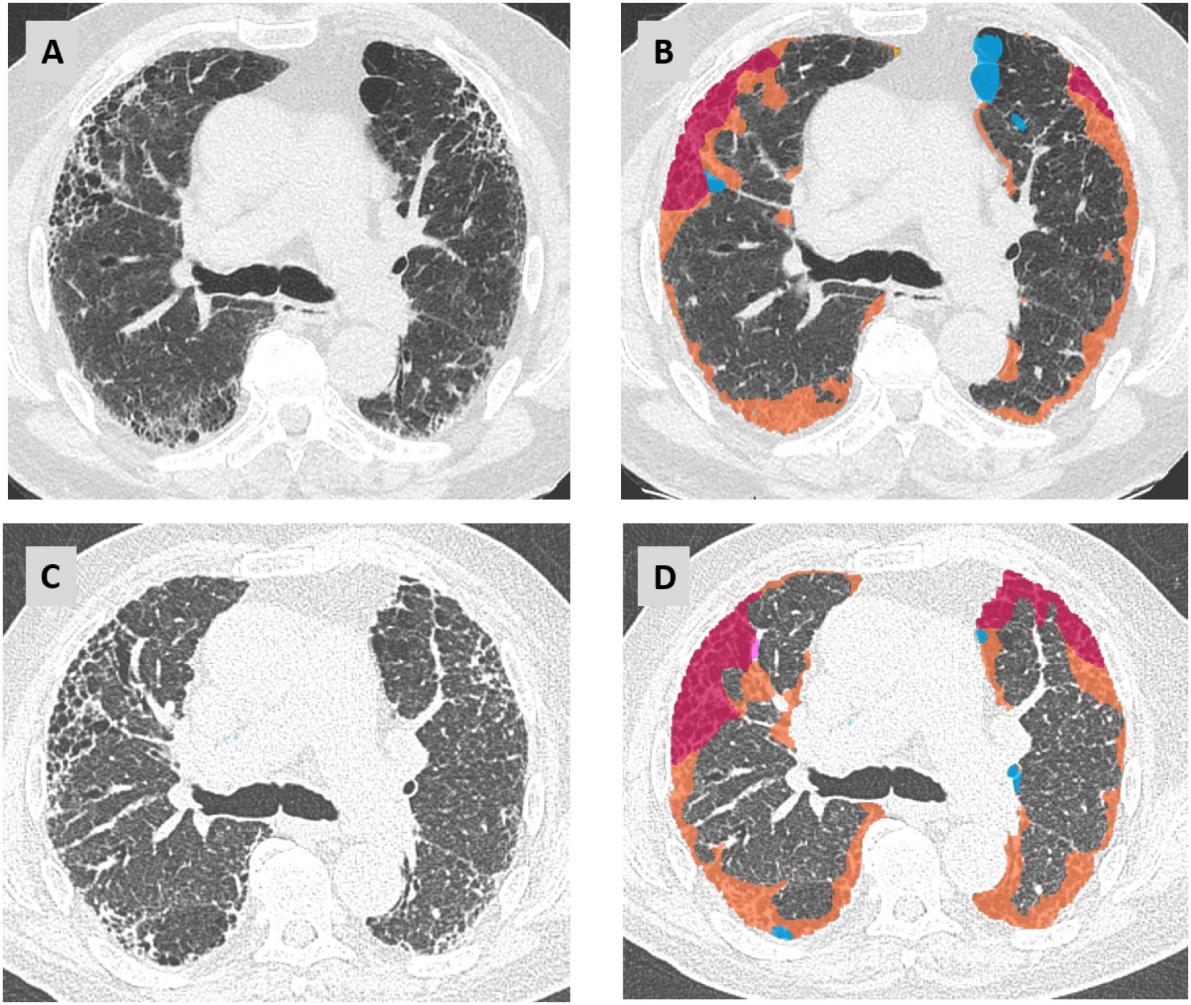
Example of a commercially available AI-based QCT algorithm (AVIEW Lung Texture, Coreline Soft, Seoul, Korea). Serial CT scans from a patient with RA-ILD exhibiting a usual interstitial pneumonia (UIP) pattern. Axial images at the level of the carina from 2019 are shown as native (A) and with QCT overlay (B), highlighting fibrotic changes with honeycombing (pink) and reticulations (orange). Paraseptal emphysema is marked in blue. Follow-up scans from 2022 (C and D) demonstrate progression of fibrosis with a slight increase in honeycombing in the right upper lobe.

In a recent deep learning-based segmentation in the prospective study of IPF patients enrolled in the PROFILE cohort, biomarkers such as reduced lung volume, increased fibrosis volume and increased vascular volume were associated with a reduced 2-year progression-free survival. Furthermore, serial lung and fibrosis volume changes were shown to be associated with differential survival.^44^ In a study by Walsh et al a deep learning-based UIP probability (SOFIA [Systematic Objective Fibrotic Imaging Analysis Algorithm]-UIP) provided enhanced outcome prediction in patients with progressive fibrotic lung disease compared to expert radiologist evaluation or guideline-based histologic pattern analysis.^45^ In another study based on deep learning-based segmentation of HRCT features, CT based markers were identified, that could predict both progressive disease and mortality.^44^ One example of a commercially available QCT algorithm is showed in Figure 6.

Despite extensive research, QCT has not yet been widely adopted in routine clinical practice. QCT measurements are highly sensitive to CT acquisition parameters, including slice thickness, reconstruction algorithms, and radiation dose, making standardization across institutions challenging. Variability in inspiratory effort, as well as confounding factors such as acute exacerbations, pneumonia, or pulmonary oedema, can further affect the accuracy and consistency of QCT-derived metrics. Additionally, most QCT studies to date are based on retrospective or observational data, with limited prospective validation, raising concerns about reproducibility and generalizability.^46^^,^^47^ Another significant barrier is the limited transparency—or “explainability”—of many machine learning models, which are often perceived as “black boxes.” This lack of interpretability makes clinicians hesitant to rely on AI-generated predictions for critical decision-making. For QCT tools to be integrated into everyday clinical workflows, future research must focus on establishing robust, reproducible imaging biomarkers and ensuring models are both interpretable and validated across diverse patient populations. Only with these advancements can QCT realize its potential as a reliable tool for predicting and monitoring progression in pulmonary fibrosis.^34^

## Management of PPF

A detailed overview of the management of PPF is beyond the scope of this article and will therefore only be discussed briefly here. Historically, 2 antifibrotic drugs, nintedanib and pirfenidone were approved for the treatment of IPF.^48–50^ However, treating patients with PPF remained challenging due to limited validated treatment options. Given that PPF shares phenotypic features with IPF, studies hypothesized that antifibrotic therapies might provide similar benefits. The INBUILD trial marked a significant breakthrough in PPF treatment by investigating nintedanib’s efficacy in progressive fibrosis beyond IPF.^51^ The trial demonstrated meaningful reductions in forced vital capacity (FVC) decline across patient subgroups. This effect was noted in patients regardless of the presence of either a UIP pattern on HRCT (61% relative reduction) or non-UIP pattern (49% relative reduction). Furthermore, the effect was consistent among varying underlying ILD diagnoses.^52^ Given these results, nintedanib was recently approved for use in patients with PPF. The RELIEF trial was a multicentre, double-blinded prospective trial in Germany evaluating the use of pirfenidone in PF-ILD patients with underlying aetiologies including CTD-ILD, f-HP, iNSIP, and asbestos-related fibrotic lung disease.^53^ Although prematurely terminated due to slow recruitment, the results of this trial suggest that in patients with PPF with progressive disease despite conventional therapy, pirfenidone therapy can attenuate further decline in FVC and disease progression and is currently available for off-label use for this indication in certain countries including Germany.^5^ Following these encouraging results, several drugs with potential antifibrotic properties are being tested in parallel phase III trials for patients with IPF or PPF.

## Conclusion and future perspectives

Progressive pulmonary fibrosis represents a clinically significant phenotype of fibrosing ILDs, with radiology playing a central role in early identification and monitoring. As newer therapies emerge, the landscape of ILD diagnosis and management is increasingly shifting toward early detection of disease progression and the identification of reliable imaging biomarkers to predict clinical behaviour. Radiology now plays a more central and proactive role in this paradigm. To realize the full potential of quantitative CT, future research must prioritize international collaboration aimed at standardizing imaging protocols, validating robust biomarkers, and facilitating the seamless integration of QCT into clinical practice.
